# Transcriptomic and ChIP-seq Integrative Analysis Identifies KDM5A-Target Genes in Cardiac Fibroblasts

**DOI:** 10.3389/fcvm.2022.929030

**Published:** 2022-07-01

**Authors:** Yiyao Jiang, Xu Zhang, Ting Wei, Xianjie Qi, Isah Amir Abba, Nana Zhang, Yao Chen, Ran Wang, Chao Shi

**Affiliations:** ^1^Department of Cardiovascular Surgery, The First Affiliated Hospital of Bengbu Medical College, Bengbu, China; ^2^Department of Cardiovascular Surgery, Tianjin First Center Hospital and NanKai University, Tianjin, China; ^3^Department of Cardiovascular Medicine, The First Affiliated Hospital of Bengbu Medical College, Bengbu, China; ^4^Department of Emergency Medicine, The First Affiliated Hospital of Bengbu Medical College, Bengbu, China

**Keywords:** KDM5A, cardiac fibrosis, differentially expressed genes, integrative bioinformatics analysis, cardiac fibroblasts

## Abstract

Cardiac fibrosis is a common pathological feature in cardiac remodeling. This study aimed to explore the role of KDM5A in cardiac fibrosis via bioinformatics analysis. Cardiac fibroblasts (CFs) were harvested and cultured from 10 dilated cardiomyopathy (DCM) patients who underwent heart transplantation. Western blotting was applied to verify that KDM5A is regulated by angiotensin II (Ang II) via the PI3k/AKT signaling pathway. The differentially expressed genes (DEGs) were analyzed by transcriptomics. ChIP-seq and ChIP-qPCR were used to identify the genes bound by KDM5A. In integrative analysis, weighted gene coexpression network analysis (WGCNA) was performed to identify highly relevant gene modules. Gene Ontology (GO) and Kyoto Encyclopedia of Genes and Genomes (KEGG) pathway enrichment analyses were performed for the key genes in modules. The STRING database, Cytoscape, and MCODE were applied to construct the protein–protein interaction (PPI) network and screen hub genes. To verify the expression of DEGs regulated by KDM5A, Western blotting and immunofluorescence were performed in myocardial tissue samples. Immunofluorescence verified the vimentin positivity of CFs. Ang II upregulated the expression of KDM5A in CFs via the PI3K/AKT signaling pathway. GO analysis of DEGs indicated that regulation of vasoconstriction, extracellular region, and calcium ion binding were enriched when KDM5A interfered with CPI or Ang II. KEGG analysis of the DEGs revealed the involvement of ECM-receptor interaction, focal adhesion, PI3K-Akt signaling pathway, cell adhesion, and arrhythmogenic right ventricular cardiomyopathy pathways. Three hub genes (IGF1, MYH11, and TGFB3) were identified via four different algorithms. Subsequent verification in patient samples demonstrated that the hub genes, which were regulated by KDM5A, were downregulated in DCM samples. KDM5A is a key regulator in the progression of cardiac fibrosis. In this successful integrative analysis, IGF1, MYH11, and TGFB3 were determined to be coordinately expressed to participate in cardiac fibrosis.

## Introduction

Cardiac fibrosis is a common pathological feature in cardiac remodeling that leads to heart failure (1). Cardiac fibroblasts (CFs), an abundant cell type in the heart, play a key role in the onset and development of cardiac fibrosis (2, 3). When CFs are activated, they become more specialized in the secretion of extracellular matrix (ECM) proteins and collagens (4). The excess accumulation of ECM impairs cardiac contractile function and increases arrhythmogenicity (5). Because of its crucial roles in the cardiac fibrotic process, CF activation, as an important therapeutic target, has gained substantial attention in this field (6). However, the current treatment options for CFs are limited, and there is a clear need to identify novel regulators of CFs to facilitate the development of better therapeutics.

The regulation of CF proliferation and migration by the PI3K/AKT signaling pathway has been confirmed widely in previous studies (7, 8). As an effector mediated by the PI3K/AKT signaling pathway, lysine-specific demethylase 5A (KDM5A) is responsible for driving multiple human diseases, particularly cancers (9, 10). Moreover, KDM5A is associated with heart failure, congenital heart disease, and arrhythmogenic right ventricular cardiomyopathy (11–13). However, it is currently unknown whether PI3K/AKT/KDM5A regulates the proliferation of CFs and, if it does so, whether this regulation is related to cardiac fibrosis. Furthermore, KDM5A can act as either a transcriptional activator or repressor to regulate gene expression (14). The comprehensive gene expression pattern in CFs is poorly understood.

Recent advances in bioinformatics technologies have allowed significant improvements in the discovery of pathogenic genes of cardiovascular diseases. A large amount of gene data has been accumulated, and sophisticated methods for analyzing data have become crucial to explain the causes of disease (15). In this study, we screened differentially expressed genes (DEGs) in response to the regulation of KDM5A. Weighted gene coexpression network analysis (WGCNA) was used to build a gene coexpression network, to screen important modules and to filter the key genes. Gene Ontology (GO) and Kyoto Encyclopedia of Genes and Genomes (KEGG) pathway enrichment analyses were performed for the key genes in modules of clinical interest. This paper provides a novel bioinformatic analysis of KDM5A in CFs and predicts the molecular mechanism of cardiac fibrosis.

## Materials and Methods

### Harvest Myocardial Tissue

In a previous study, we carried out 103 heart transplantations (16). During the operations, myocardial tissues were obtained from 10 DCM patients. Four ventricular samples were used for cell culture. Six samples were used to verify the results of the bioinformatics analysis. The left atrial appendage (LAA), used as the control sample, was obtained during a previous valve replacement surgery (17).

### Isolation and Culture of Primary Cardiac Fibroblasts

Isolation of human primary CFs was performed as previously reported. Briefly, myocardial tissue was minced to 1 mm in cold phosphate-buffered saline. Minced tissue was subsequently digested with buffer containing collagenase II (Sigma–Aldrich, USA) and trypsin (Solarbio Life Sciences, Beijing) with constant stirring at 37 °C for 60 min. The supernatants were spun to collect cells. Then, cells were resuspended in DMEM/F12 (Gibco, MA), plated into dishes and incubated for 2 hr. The supernatant was discarded, and the dishes were replenished with fresh medium. CFs were incubated at 37 °C in a humidified atmosphere of 5% CO_2_ and grown to 70–80% confluence. Cells at passages 2 to 3 were used in experiments.

### CCK8 Assay

Cell viability was evaluated by a Cell Counting Kit-8 (CCK-8, Abbkine, U.S.A.); 96-well plates were seeded with 3 × 10^3^ CFs per 100 μl, and the cells were cultured as previously described for 24–48 h (18). After the medium was exchanged, CCK-8 reagent was added, followed by incubation for 60 min and spectrophotometry to assess the OD_450_ value.

### Immunofluorescence

CFs were fixed with PBS/paraformaldehyde (4%) for 10 min and permeabilized with PBS/Triton X-100 (0.5%) for 5 min. Then, they were incubated with anti-vimentin antibody (1:50, Abcam, ab92547) at 4 °C overnight. Cy3-conjugated secondary antibodies (Invitrogen, A10522) were used at 1:100. Nuclei were counterstained with DAPI (Sigma–Aldrich, USA).

The procedure for immunofluorescence staining of myocardial tissue sections was as described in our previous study (17). The sections were incubated with vimentin (1:500, Abcam, ab92547), KDM5A (1:2000, Abcam, ab194286), IGF1 (1:400, Abcam, ab223567), MYH11 (1:1000, Abcam, ab82541), and TGFB3 (1:1000, Abcam, ab15537) overnight at 4 °C. Then, the sections were incubated with Alexa Fluor 488 donkey anti-mouse immunoglobulin G (Abcam, ab150105), Alexa Fluor 594 donkey anti-rabbit immunoglobulin G (Abcam, ab150156) or Alexa Fluor 633 donkey anti-goat immunoglobulin G (Invitrogen, A-21082). The nuclei were counterstained with DAPI (2.5 μg/ml in PBS; Molecular Probes).

### Western Blotting

Cells and ventricular tissue were lysed with RIPA buffer (Sigma–Aldrich, USA), and the protein concentrations of the lysates were determined using BCA Protein Assay Reagent (Pierce Biotechnology, USA). Total protein samples (40 μg) were separated electrophoretically, transferred to a PVDF membrane and probed using monoclonal primary antibodies against PI3K (1:5000, Abcam, ab139307), p-PI3K (1:1000, Abcam, ab32089), AKT (1:1000, Abcam, ab179463), p-AKT (1:1000, Cell signaling, 4060s), KDM5A (1:5000, Abcam, ab194286), IGF1 (1:1000, Abcam, ab223567), MYH11 (1:1000, Abcam, ab82541), TGFB3 (1:1000, Abcam, ab15537), and β-actin (1:500, Abcam, ab5694), followed by an HRP-conjugated secondary antibody (1:5000; Abbiotec, USA). Bands were exposed using an ECL kit (Bio–Rad, USA) and analyzed using Image-Pro Plus software. LY294002 (20 μmol/L, Cell signaling, 9901s) is an inhibitor of PI3K, and ARQ-092 (10 μmol/L, Abcam, ab235550) is an inhibitor of AKT.

### Transcriptomic Analysis

Transcriptomic analysis was performed according to our previous study. Briefly, total RNA was extracted from CFs using TRIzol reagent (Invitrogen, Burlington, ON, Canada) and digested with DNase I (Takara, Dalian, China) according to the manufacturer's protocol. Next, Oligo(dT) magnetic beads were used to isolate mRNA from the total RNA. By mixing with fragmentation buffer, the mRNA was then broken into short fragments. cDNA was synthesized using the mRNA fragments as templates. The short fragments were purified and resolved with EB buffer for end repair and single nucleotide A (adenine) addition and then connected with adapters. Suitable fragments were selected for PCR amplification as templates. During the quality control steps, an Agilent 2100 Bioanalyzer (Agilent Technologies, Redwood City, CA, USA) and ABI StepOnePlus Real-Time PCR System (Life Technologies, Grand Island, NY, USA) were used for quantification and qualification of the sample library. Each cDNA library was sequenced in a single lane of the Illumina HiSeqTM 2000 system using paired-end protocols according to the manufacturer's instructions at the Beijing Genomics Institute (BGI) (Shenzhen, China). For transcriptomic analysis, three biological replicates were performed.

### ChIP-seq and ChIP-qPCR Assay

Chromatin was prepared from fixed CFs (stimulated with 1 μmol/L CGRP, 2 h) and sonicated to fragments ranging in size from 200 to 800 bp. Approximately 2 × 10^7^ cell equivalents were used for each immunoprecipitation. ChIP was performed as described previously (19) using anti-KDM5A antibody or a control rabbit IgG. 20 μL of the chromatin solution was saved as an input control.

DNA samples were end-repaired, A-tailed, and adaptor-ligated. Then, 100-500 bp fragments were size-selected using AMPure XP beads. The final size of the library was confirmed by an Agilent 2100 Bioanalyzer. Samples were diluted to a final concentration of 8 pmol/L, and cluster generation was performed on the Illumina cBot. Massively parallel was performed on an Illumina HiSeq 4000. ChIP-seq was done with three biological replicates.

Aligned reads were submitted for peak calling of ChIP regions using MACS. Statistically significant ChIP-enriched regions (peaks) were identified by comparison of IP vs. input or comparison to a Poisson background model, using a *P*-value threshold of 10^−4^. Peaks in samples were annotated to the nearest gene using the newest UCSC RefSeq database. The annotation of peaks located within−2 kb to +2 kb of the corresponding gene transcription start site (TSS) were identified as promoter peaks. After the sequencing platform generated the sequencing images, image analysis, and base calling were performed using Integrative Genomics Viewer (IGV) software (Version 2.13).

For ChIP-qPCR, the input DNA pulled out from ChIP was diluted ten times with ChIP immunoprecipitation buffer (0.01% SDS, 1% Triton X100, 2 mM EDTA, 50 mM Tris–HCl pH 8.0, 150 mM NaCl, and protease inhibitor cocktail) before qPCR, while the IgG and KDM5A groups were added with their ChIP grade antibodies using the suggested concentration from the manufacturers. After purification, immunoprecipitated DNA was detected by qPCR and normalized with input DNA. The sequences of the primers for ChIP-qPCR are listed in Supplementary Table S1.

### Bioinformatics Analysis

Integrative analysis of ChIP-Seq and transcriptomic data was performed with the R package as described in a previous study (20). Weighted gene coexpression network (WGCNA) analysis was conducted with the R package. The lowest threshold power for the scale-free topology fit index was 0.9. The ME-Diss Thresh was set at 0.1 to merge similar modules.

After importing the MEdarkgray model DEGs, the protein–protein interaction network was predicted by STRING. The minimum required interaction score was medium confidence (0.400). In this study, the PPI network was constructed and analyzed by the STRING database (http://string-db.org, Version 11.5). Cytoscape (https://cytoscape.org, Version 3.90) was used for visualization analysis, and node connectivity was calculated with the plug-in Molecular Complex Detection (MCODE) (Version 1.5.0) to screen for the central node of the network. Genes corresponding to the central node were considered core genes. An MCODE score > 3 and number of nodes >4 were considered threshold values.

Functional inference analysis of the network was performed with the Gene Ontology (GO) database (http://geneontology.org/docs/introduction-to-go-resource/, version https://doi.org/10.5281/zenodo.6363634), specifically, the cellular component (CC) and biological pathway (BP) subsets. Kyoto Encyclopedia of Genes and Genomes (KEGG) (https://www.genome.jp/kegg/docs/relnote.html, Version 101.0) analysis can be used to highlight specific pathways and link genomic information with higher-order functional information. Gene set enrichment analysis (GSEA) is a computational method that can perform GO and KEGG analysis on a given gene list. Metascape provides a comprehensive gene list annotation and analysis resource. In this study, GO and KEGG analyses of MEdarkgray model DEGs were performed by GSEA (Version 4.2) and Metascape (http://metascape.org/gp/index.html#/main/step1, Version 3.5).

Hub genes were identified using the degree, edge percolated component (EPC), maximal clique centrality (MCC), and maximum neighborhood component (MNC) algorithms. We applied the four different algorithms and then made Venn diagrams of the results. The overlaps of hub genes identified by different algorithms are performed by Circos (http://circos.ca/software/download/circos, Version 0.69-9). After confirming the common hub genes, the relationships between gene products and heart diseases were analyzed by the comparative toxicogenomics database (CTD) [http://ctdbase.org/, released March 2, 2022 (16729)].

### Statistical Analysis

The analyses in this study were performed using Perl, R (version 4.02), SPSS 23.0 (SPSS Inc., Chicago, IL, USA), and GraphPad Prism 9.0 [GraphPad Software Inc., La Jolla, USA). The DEGs were screened by the R package. The cutoff criteria were P value < 0.05 and |Log Fold Change (FC)] |≥ 2. The Benjamini and Hochberg method (false discovery rate) was used to obtain adjusted *P*-values.

## Results

### Ang II Upregulated the Expression of KDM5A via the PI3K/AKT Signaling Pathway

Immunofluorescence staining showed that CFs expressed vimentin on their surface (Supplementary Figure S1A). CCK8 was used to analyze the effect of the concentration of Ang II on CF proliferation. The results revealed that the cell proliferation ability was significantly higher under the concentration of 1 × 10^−7^ mol/L at 24 and 48 hours, respectively (Supplementary Figure S1B). Therefore, this concentration (1 × 10^−7^ mol/L) was applied in this study.

**Figure 1:**
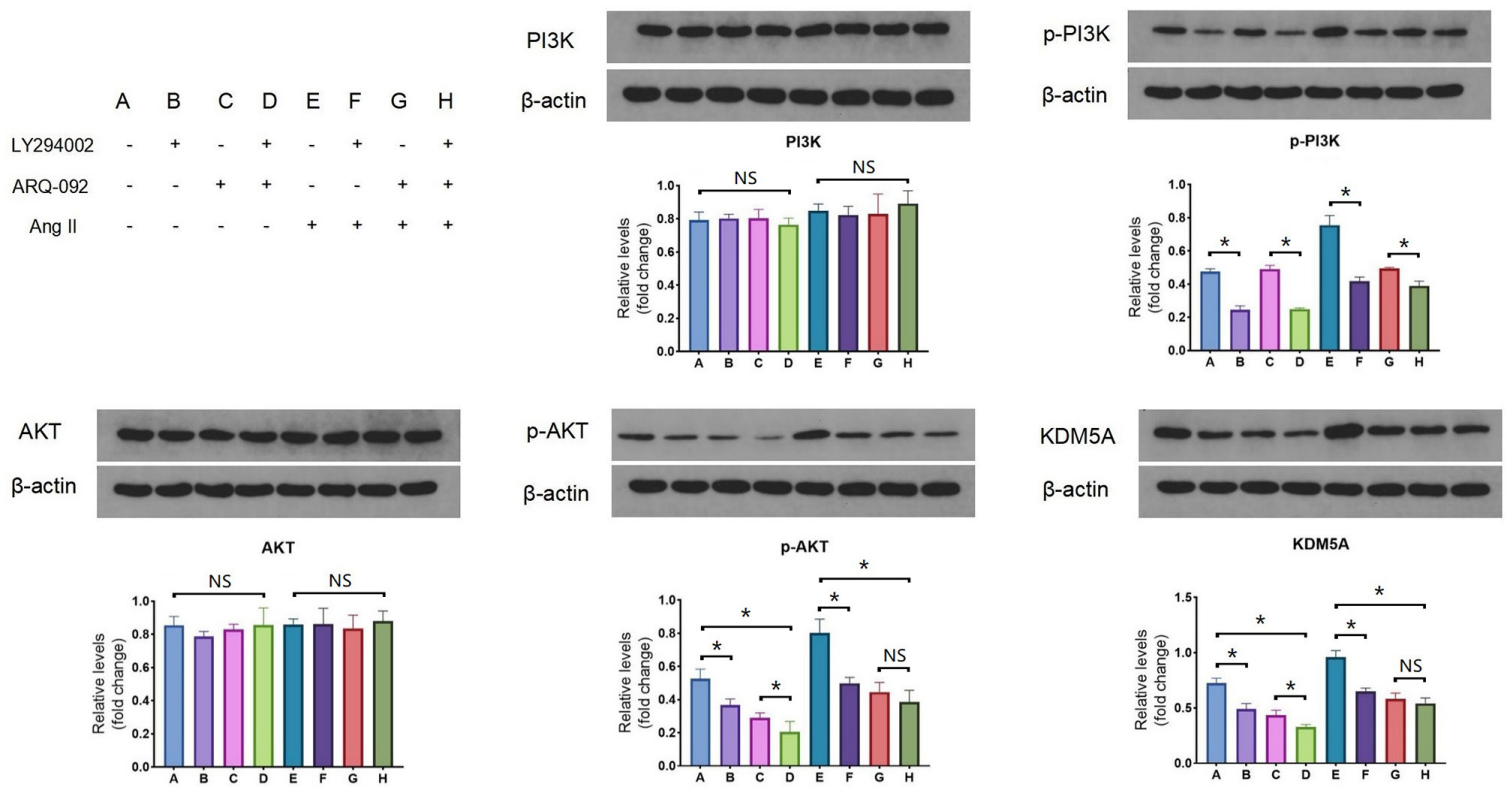
Western blotting results of PI3K, p-PI3K, AKT, p-AKT and KDM5A protein expression in CFs treated with Ang II, LY294002, and ARQ-092. LY294002 (20 μmol/L) is an inhibitor of PI3K; ARQ-092 (10 μmol/L) is an inhibitor of AKT. Beta-actin was used as the reference gene. Each data point was obtained from three replicate experiments. *P < 0.05.

To determine whether Ang II upregulated the expression of KDM5A via the PI3K/AKT signaling pathway, Western blotting was performed. Although no changes in the total protein level of PI3K were observed after 24 h of LY294002 treatment, there was a significant decrease in the level of phosphorylated PI3K. Phosphorylation of total Akt in CFs was also altered by ARQ-092 treatment compared to the control. However, we found that the culture of CFs with 1 × 10^−7^ mol/L Ang II induced a stable increase in KDM5A. Taken together, these results suggest that Ang II upregulated KDM5A expression in CFs via the PI3K/Akt signaling pathway (Figure 1).

### Transcriptomic Study

We used RNA-Seq for the transcriptomic study of CFs incubated with Ang II or CPI (an inhibitor of KDM5A) (21). Compared to CFs, in the Ang II group, 2744 DEGs were upregulated and 2618 DEGs were downregulated. Similarly, 2577 DEGs were upregulated and 2544 DEGs were downregulated in CPI. These DEGs are involved in well-defined functions, such as regulation of phospholipase C activity, negative regulation of hemostasis, muscle contraction, cellular extravasation, parathyroid hormone receptor activity, and cell-matrix adhesion. The raw transcriptome data have been submitted to PubMed (BioProject ID: PRJNA797118).

### ChIP-seq and ChIP-qPCR Study

**Figure 2:**
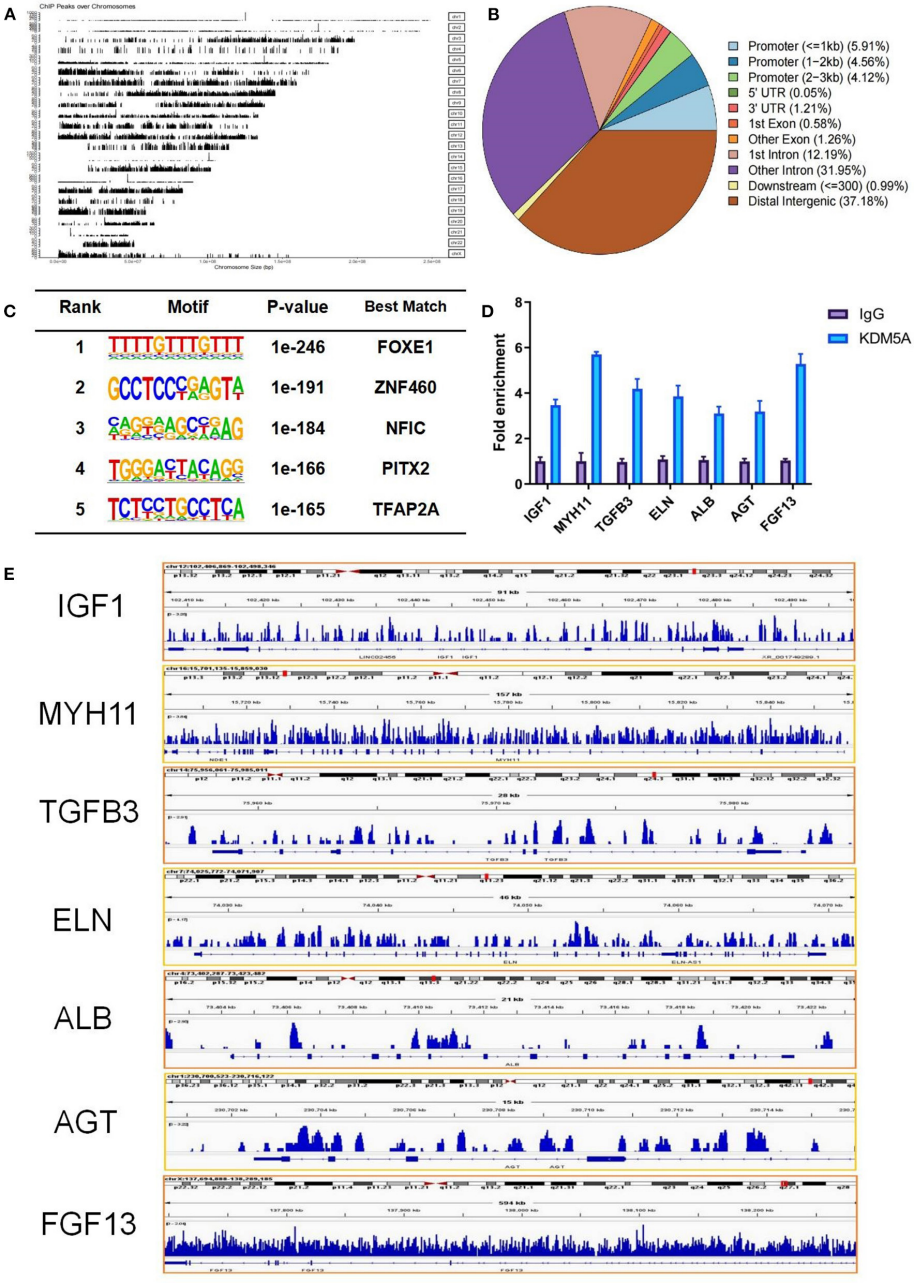
**(A)** From ChIP-seq, a total of 3.2 × 10^7^ reads mapped to the reference genome. **(B)** The peaks were distributed in the promoter, UTR, exon, intron, downstream, and distal intergenic regions. **(C)** Characteristic analysis of KDM5A protein binding motif sequence by Homer. **(D)** IGF1, MYH11, TGFB3, ALB, AGT, FGF13, and ELN enrichment in KDM5A binding sites of the DNA samples in CFs as assessed by ChIP-qPCR. **(E)** Enriched peaks were identified using the ChIP-seq data via IGV. Each data point was obtained from three replicate experiments.

To determine the genomic regions bound by KDM5A, we performed ChIP-seq. Upon completion of sequencing, the percentage of reads that passed the internal quality filtering was >93.4%. A total of 3.2 × 10^7^ reads were mapped to the reference genome. A total of 13152 peaks were detected, and nearly 13173 genes were near the peaks. The peaks were distributed in the promoter (14.59%), UTR (1.26%), exon (1.84%), intron (44.14%), downstream (0.99%), and distal intergenic regions (37.18%). Characteristics of KDM5A protein binding motif sequences were analyzed by Homer (Version 4.11). The best match transcription factors, such as FOXE1, ZNF460, NFIC, PITX2, and TFAP2A, were related to cell growth and migration, cell proliferation, transcription factor activation, and DNA replication. ChIP-qPCR results showed that IGF1, MYH11, TGFB3, ELN, ALB, AGT, and FGF13 had a high enrichment in KDM5A binding sites of the DNA samples. These genes regulated by KDM5A were displayed by IGV (Figure 2).

The top 5 GO terms enriched among these genes were cell morphogenesis involved in neuron differentiation, regulation of neuron projection development, axon development, plasma membrane protein complex, and actin cytoskeleton. The top 5 KEGG pathways were the phospholipase D signaling pathway, cholinergic synapse, calcium signaling pathway, focal adhesion, and Wnt signaling pathway (Supplementary Figure S2).

### Identification of DEGs

**Figure 3:**
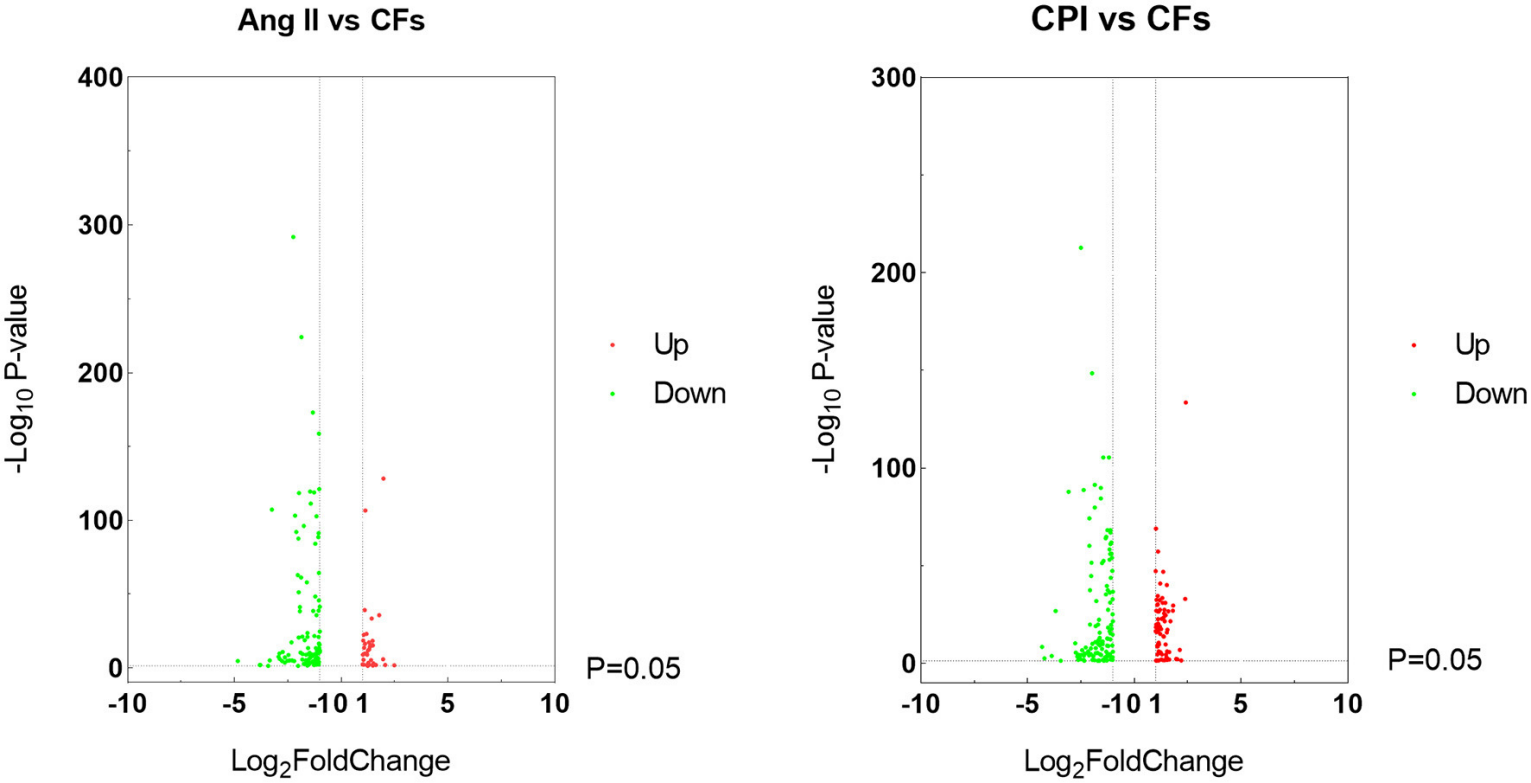
Volcano plots of the genes that are DEGs in Ang II vs. CFs and CPI vs. CFs.

As Figure 3 shows, integrative analysis of transcriptomic and ChIP-seq data via WGCNA identified 119 DEGs bound by KDM5A in Ang II-treated compared with untreated CFs, including 101 downregulated DEGs and 18 upregulated DEGs. When compared with untreated CFs, a total of 133 DEGs were detected in CPI-treated cells. Among these DEGs, 37 were upregulated genes, and 96 were downregulated genes.

### PPI Network Analysis

**Figure 4:**
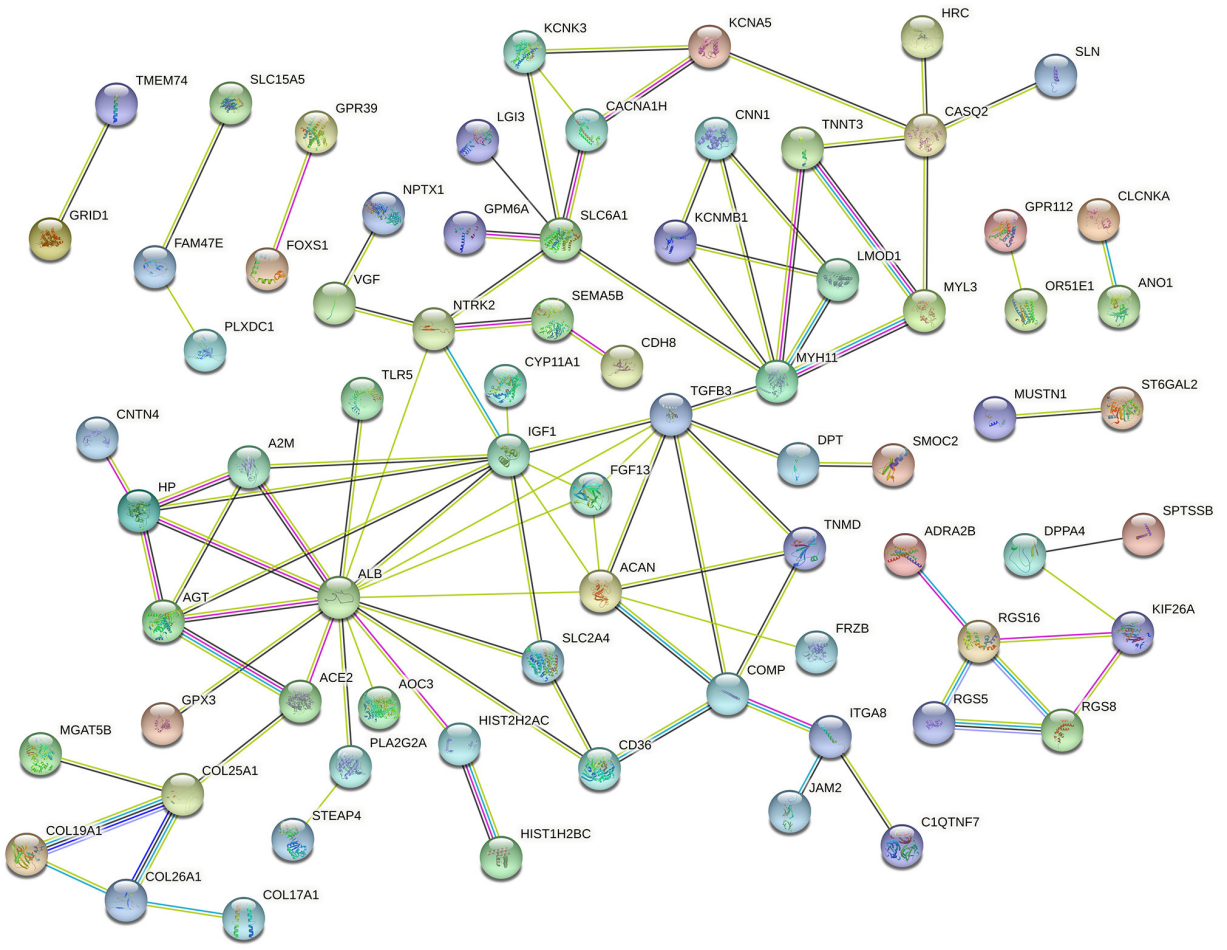
Protein–protein interaction network of DEGs in Ang II vs. CFs.

The PPI network of the DEGs was constructed via the STRING online database and analyzed by Cytoscape software. The 119 DEGs identified between Ang II-treated and untreated CFs were imported into the STRING database to construct the PPI network, and 73 DEGs were incorporated into the PPI network complex (Figure 4). MCODE in Cytoscape was used to calculate the degree, edge percolated component (EPC), maximal clique centrality algorithm (MCC), and maximum neighborhood component (MNC) of each protein in the PPI network. We detected a significant cluster containing SLC6A1, NTRK2, IGF1, ALB, TGFB3, MYH11, ITGA8, CACNA1H, AGT, SMOC2, FGF13, ACAN, FRZB, COMP, and TNMD (Supplementary Figure S3).

**Figure 5:**
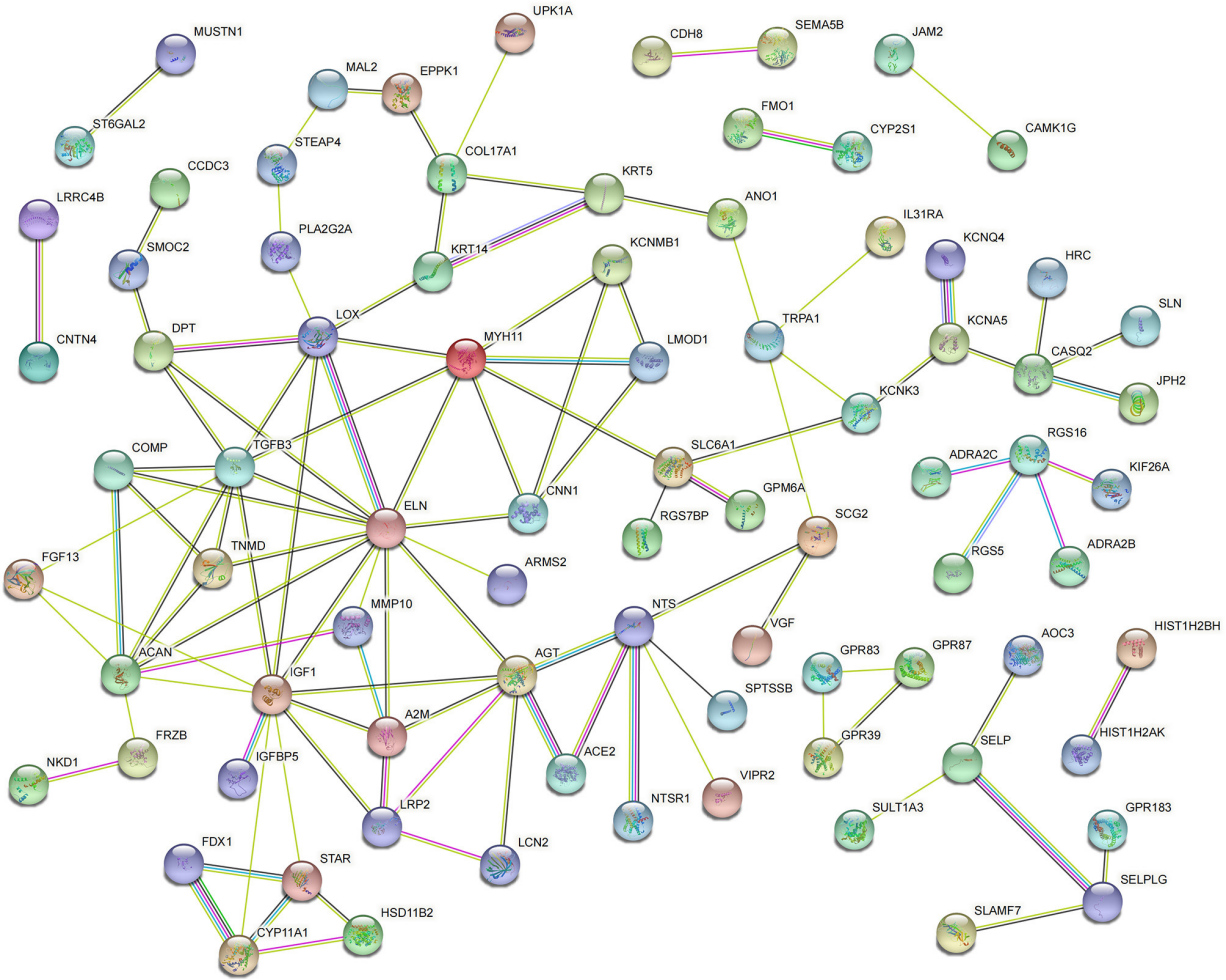
Protein–protein interaction network of DEGs in CPI vs. CFs.

For CFs treated with CPI, 82 DEGs were incorporated into the PPI network complex (Figure 5). MCODE in Cytoscape detected a significant protein cluster containing TGFB3, IGF1, MYH11, ELN, ACAN, AGT, FGF13, SLC6A1, LCN2, SELP, CNN1, COMP, TNMD, and DPT (Supplementary Figure S4).

### Identification of Hub DEGs Associated With Cardiovascular Diseases

**Figure 6:**
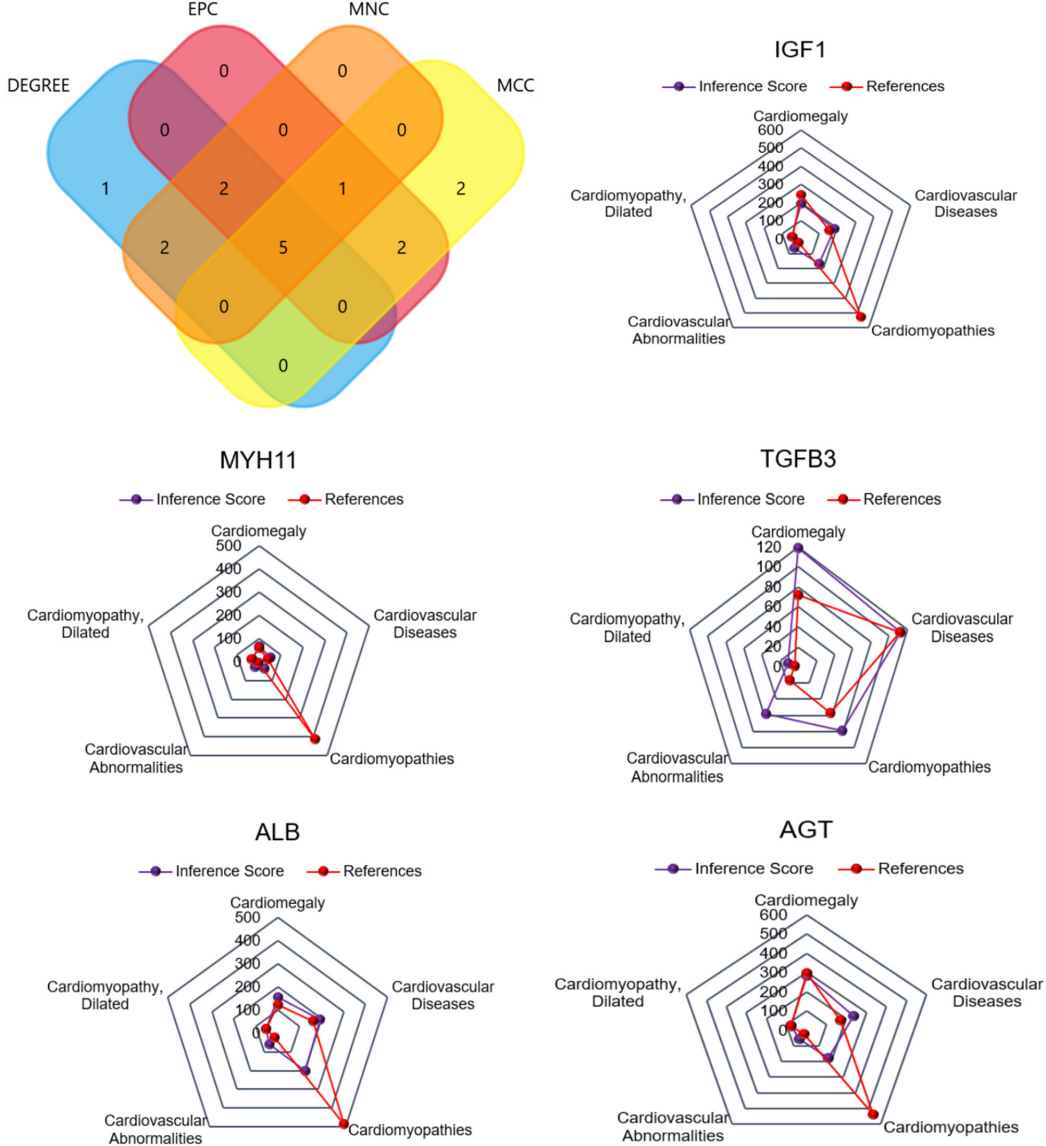
The common hub genes identified from degree, edge percolated component (EPC), maximal clique centrality algorithm (MCC), and maximum neighborhood component (MNC) analysis of Ang II vs. CFs. IGF1, MYH11, TGFβ3, ALB, and AGT were identified as related to cardiovascular disease based on the CTD database.

**Figure 7:**
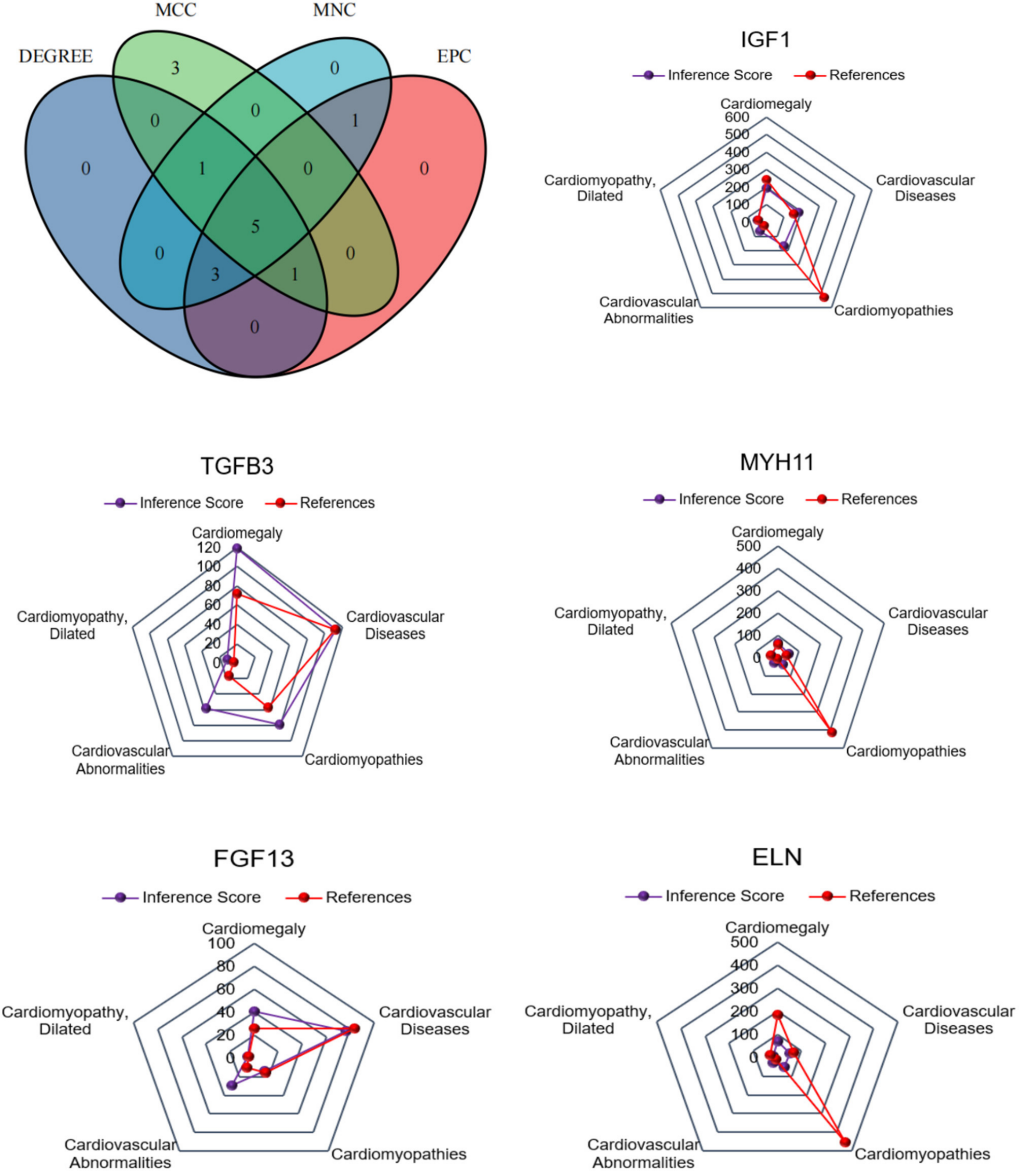
The common hub genes identified from degree, edge percolated component (EPC), maximal clique centrality algorithm (MCC), and maximum neighborhood component (MNC) in CPI vs. CFs. IGF1, MYH11, TGFβ3, FGF13, and ELN were identified as related to cardiovascular disease based on the CTD database.

For CFs treated with Ang II, a Venn diagram was constructed and revealed that 5 genes were simultaneously identified as hub genes by the four different algorithms. The CTD database showed that the common hub genes (IGF1, MYH11, TGFB3, ALB, and AGT) were targets in cardiomyopathies. The database also highlighted cardiomegaly, dilated cardiomyopathy, and cardiovascular diseases (Figure 6). For CFs treated with CPI, the Venn diagram and CTD database results were similar to those for Ang II. The hub genes were IGF1, MYH11, TGFB3, FGF13, and ELN (Figure 7).

### GO and KEGG Pathway Enrichment Analysis

**Figure 8:**
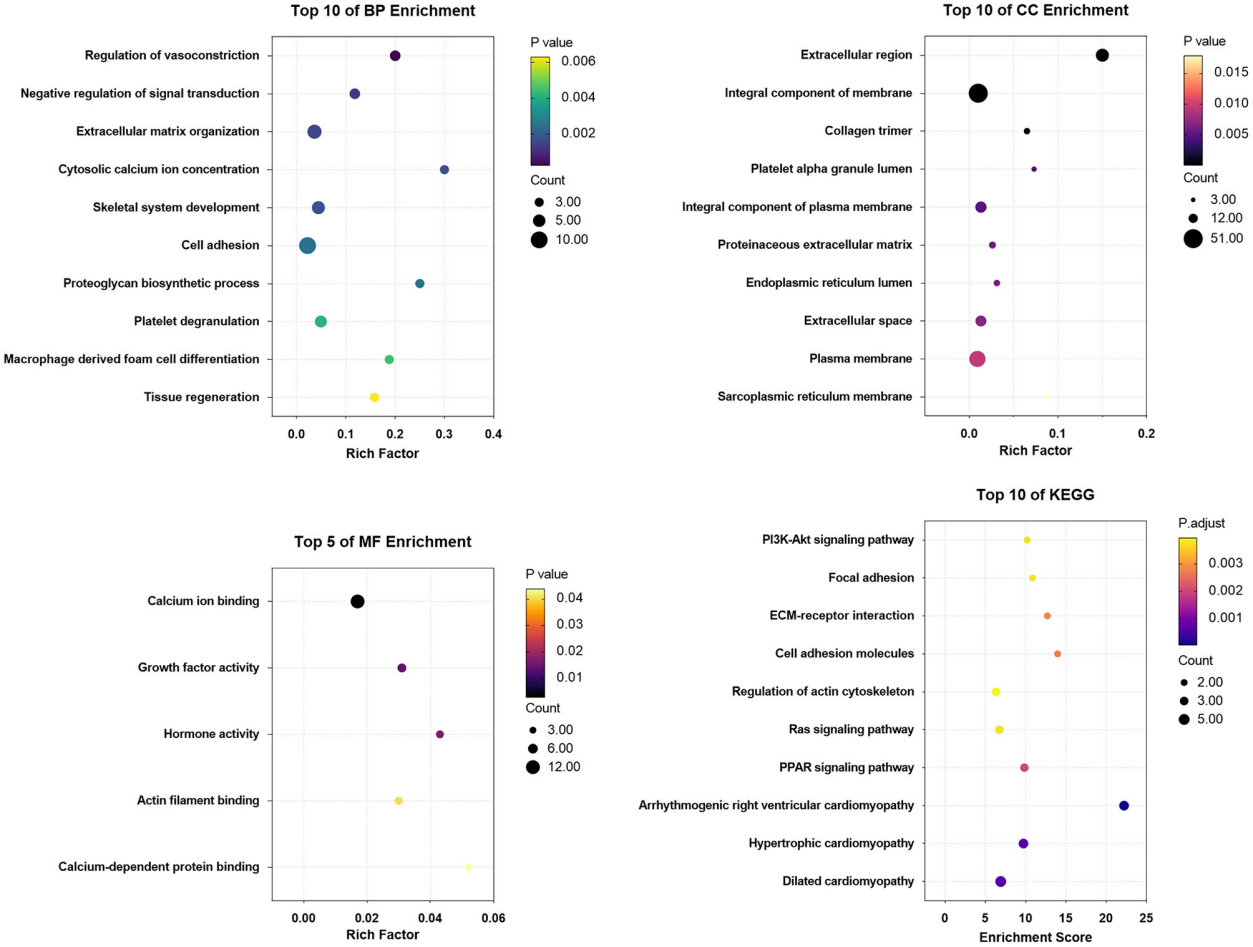
The enrichment analysis of DEGs in Ang II vs. CFs by DAVID and Metascape. Detailed information relating to changes in the CC, BP, MF, and KEGG analysis for hub genes is shown.

GO terms were divided into three functional categories: biological process (BP), cellular component (CC), and molecular function (MF). The DEGs between Ang II incubated and untreated CFs mainly participated in regulation of vasoconstriction, negative regulation of signal transduction, and extracellular matrix organization (BP categories); extracellular region, integral component of membrane, and collagen trimer (CC categories); and calcium ion binding, growth factor activity, and hormone activity (MF categories). KEGG analysis of the DEGs showed enrichment in the PI3K-Akt signaling pathway, focal adhesion, ECM-receptor interaction, etc. (Figure 8).

**Figure 9:**
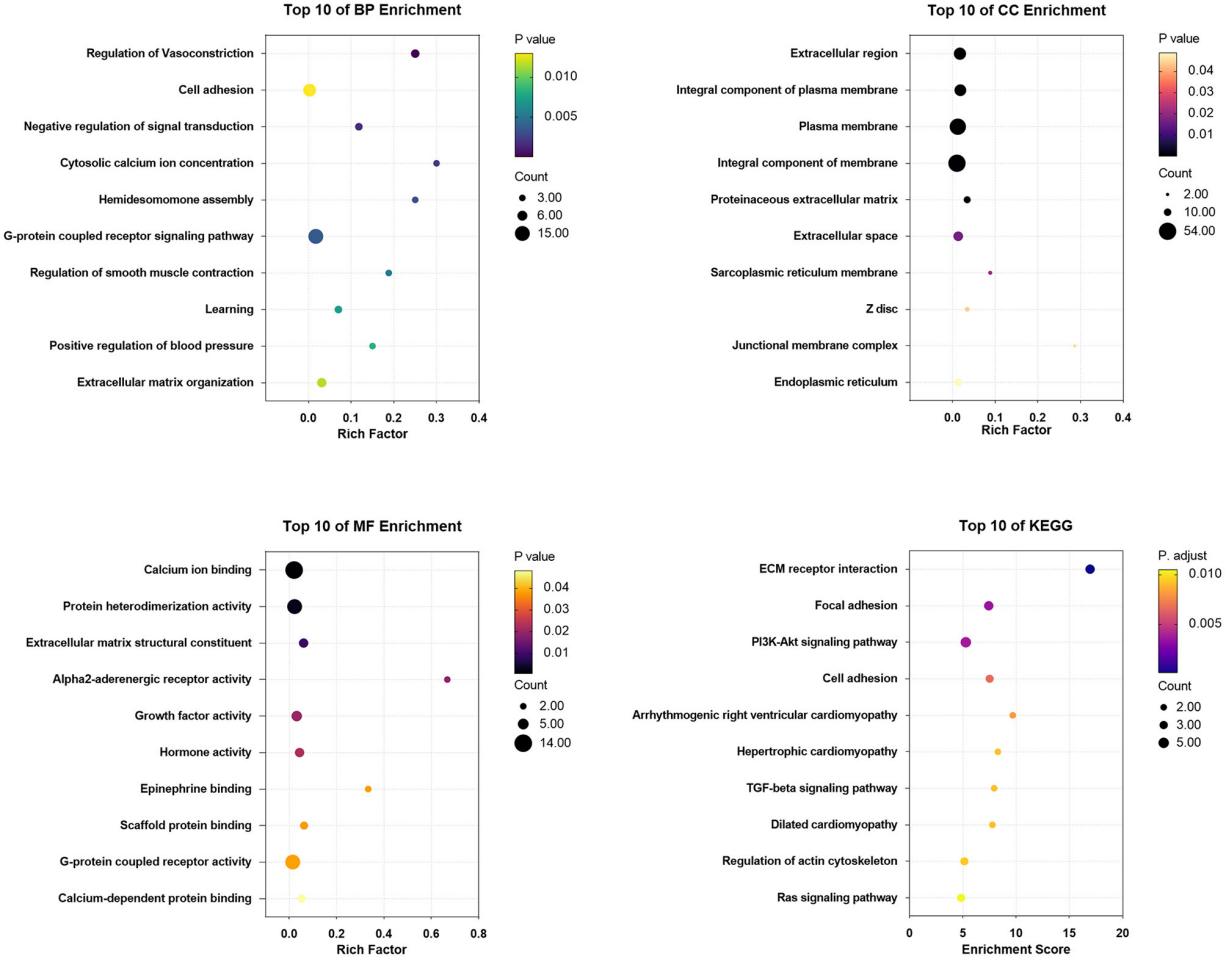
The enrichment analysis of DEGs in CPI vs. CFs by DAVID and Metascape. Detailed information relating to changes in the CC, BP, MF, and KEGG analysis for hub genes is shown.

The DEGs between CPI treated and untreated CFs were mainly involved in regulation of vasoconstriction, cell adhesion, and negative regulation of signal transduction (BP categories); extracellular region, integral component of plasma membrane, and plasma membrane (CC categories); and calcium ion binding, protein heterodimerization activity, and extracellular matrix structural constituent (MF categories). KEGG analysis of the DEGs showed enrichment in ECM-receptor interaction, focal adhesion, PI3K/Akt signaling pathway, cell adhesion, arrhythmogenic right ventricular cardiomyopathy, etc. (Figure 9).

### Verification of the Expression of KDM5A and Hub Genes

**Figure 10:**
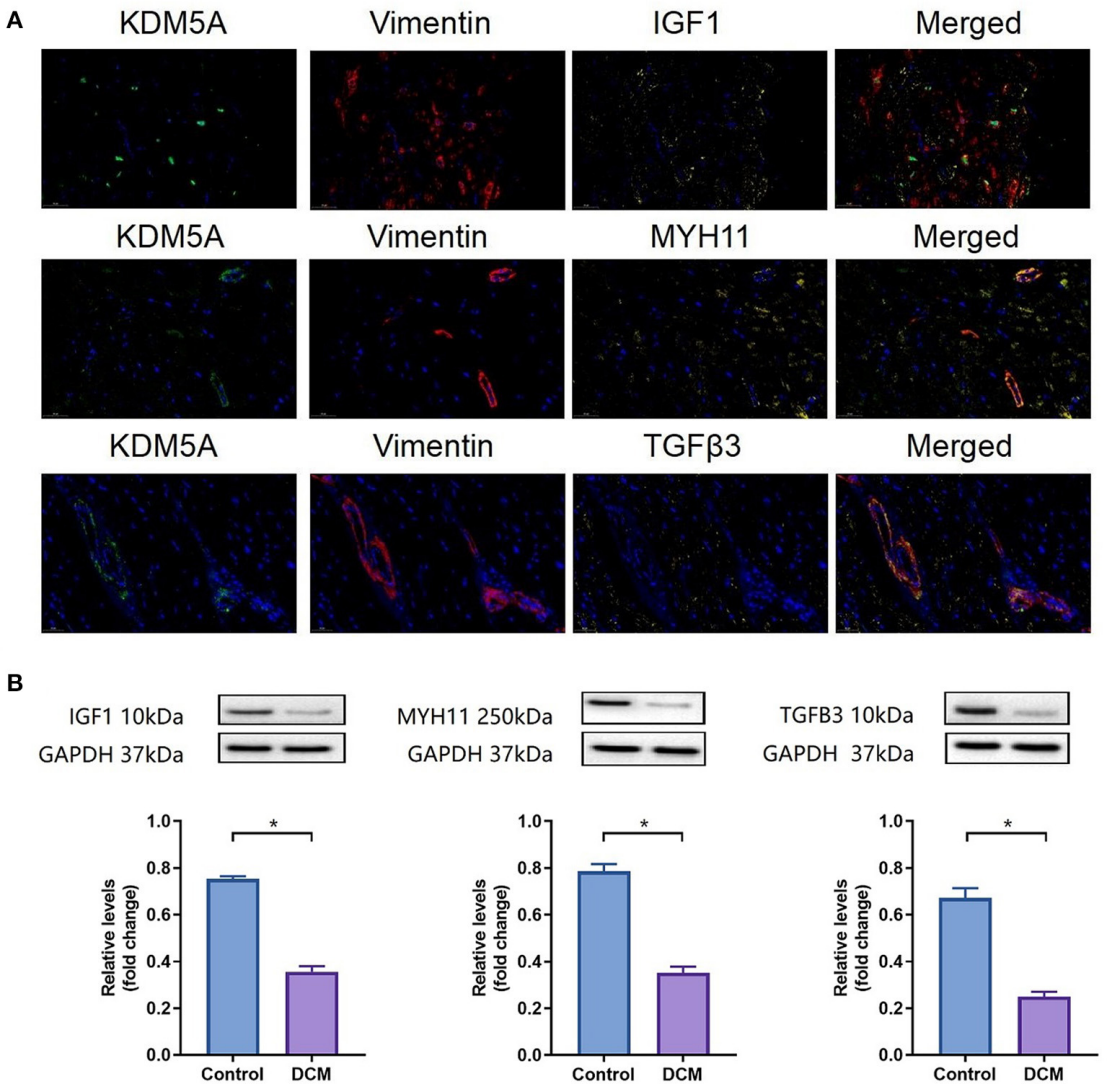
**(A)** IF staining for KDM5A, Vimentin, IGF1, MYH11, and TGFβ3 in myocardial tissue from DCM patients. DAPI was used for nuclear staining; Alexa Fluor 488 was used to detect KDM5A, Alexa Fluor 594 was used to detect Vimentin, and Alexa Fluor 633 was used to detect IGF1, MYH11, and TGFβ3. **(B)** Western blotting results of IGF1, MYH11, and TGFβ3 protein expression in DCM patients and controls. Each data point was obtained from three replicate experiments. *P < 0.05.

As shown in Figure 10, the fold change in KDM5A, IGF1, MYH11, and TGFB3 expression was significantly lower in DCM patients (*P* < 0.05). Immunofluorescence assay results corroborated this finding.

## Discussion

In this study, WGCNA of an integrative RNA-seq and ChIP-seq analysis of CFs predicted that KDM5A played a key role in regulating the expression of DEGs such as IGF1, MYH11, and TGFB3. These DEGs had a high degree of interaction in the PPI network. Further bioinformatics analysis suggested that DEGs regulated by KDM5A were mainly enriched in the PI3K-Akt signaling pathway, focal adhesions, ECM-receptor interactions, etc. These pathways are broadly involved in the progression of cardiac fibrosis.

### Role of KDM5A in Cardiac Remodeling

Ang II, a core component of the renin-angiotensin system, plays a key role in the onset and development of cardiac remodeling. Previous studies have shown that Ang II induces cardiac hypertrophy, apoptosis, and cardiac oxidative stress via the PI3K/AKT signaling pathway (22, 23). KDM5A is an AKT target, and phosphorylation of KDM5A regulates its nuclear localization and promoter occupancy (10). Thus, we speculated that the Ang II/PI3K/AKT/KDM5A axis might be associated with cardiac remodeling. Many studies have shown that KDM5A plays an important role in epigenetic modifications during the development of heart failure (11, 13). However, the role of KDM5A in cardiac fibrosis and the correlation between genes modulated by KDM5A and CF proliferation have not been reported. In this study, we predicted via integrative analysis that IGF1, MYH11, and TGFB3 could be bound to KDM5A. These DEGs are related to cardiac remodeling. This prediction provided accurate therapeutic targets for cardiac remodeling and connected with relevant research on genes that regulate the proliferation of CFs. Therefore, we confirmed that KDM5A is a hub gene in the regulation of cardiac remodeling.

### Role of DEGs in Cardiac Fibrosis

Regarding the pathways involving these DEGs, the KEGG analysis indicated enrichment in focal adhesion, ECM-receptor interaction, cell adhesion, arrhythmogenic right ventricular cardiomyopathy, hypertrophic cardiomyopathy, and dilated cardiomyopathy. These signaling pathways have been proven to be widely related to cardiac fibrosis (24–28). However, the links among these pathways have not yet been analyzed. In this study, we analyzed the PPI network of the DEGs to investigate their potential connection. The results indicate that these DEGs bound with KDM5A are involved in the progression of cardiac fibrosis via different signaling pathways. We believe that the internal interaction among DEGs represents a cooperative effect to regulate cardiac fibrosis and even cardiac remodeling.

Among the identified DEGs, IGF1, MYH11, and TGFB3 had a high degree of interaction in the PPI network and were predicted to be key genes in cardiomyopathy. IGF1, insulin-like growth factor 1, is mainly synthesized by the liver and is also produced by cardiac fibroblasts. In a study of transgenic IGF1R mice, IGF1 activated PI3K/AKT signaling pathways and regulated cardiomyocyte survival, hypertrophy, and aging (29). This result is consistent with our KEGG analysis. To accurately repair the damaged myocardium, intravenous administration of Hoechst-IGF1 was applied to activate AKT to preserve cardiac function and prevent cardiac fibrosis in a mouse model of ischemia–reperfusion (30). Additionally, Ock S and his colleagues demonstrated that low-dose IGF1 infusion markedly inhibited the proliferation and differentiation of fibroblasts to attenuate cardiac fibrosis (31). In the present study, the low expression of IGF1 in myocardium tissue, and especially fibroblasts, in DCM patients was confirmed via western blotting and immunofluorescence. MYH11 belongs to the myosin heavy chain family and is a major contractile protein in smooth muscle cells. In previous studies, mutations in MYH11 have been identified in familial congenital heart disease and thoracic aortic aneurysms and dissections (32, 33). However, the role of MYH11 in cardiomyopathy is unclear. We found that the expression of MYH11 was downregulated in DCM. Although bioinformatic analysis predicted that MYH11 is involved in the progression of DCM, the exact mechanism needs to be verified. In contrast, the role of TGFB3 in cardiovascular disease is very clear. TGFB3 deletion leads to outflow tract remodeling, ventricular fibrosis, and extracellular matrix reorganization (34). This result suggested that TGFB3 is important for cardiac development. In our study, TGFB3 was expressed at low levels in CFs and DCM. Mutation or deficiency of TGFB3 results in failure to reorganize the collagen matrix in fibroblasts. Fibrotic lesions are associated with poor contractile function and ECM response (35). Based on this evidence, these hub genes bound with KDM5A are related to cardiac fibrosis.

The mechanism of cardiac fibrosis is complex. No single gene or signaling pathway can comprehensively explain the progression of this disease. However, bioinformatic analysis is an effective method to narrow in on core genes. In our study, we confirmed that KDM5A is a crucial regulatory node in cardiac fibrosis. There is a great need for the development of therapeutic strategies that can target KDM5A to attenuate cardiac fibrosis.

Although this study included rigorous bioinformatic analysis, there are still some limitations. First, the bioinformatic identification of hub genes is not strong proof of function, and further molecular biological experiments are needed to confirm the effect of KDM5A knockout in CFs or animals. Second, the role of KDM5A, as an activator or suppressor, should be further analyzed to better understand the molecular mechanisms regulated by KDM5A in cardiac fibrosis.

## Conclusion

In summary, the present study is one of our attempts to prove that KDM5A is a key regulator in the progression of cardiac remodeling via bioinformatics technology. In this successful integrative analysis, IGF1, MYH11, and TGFB3 were determined to be coordinately expressed and participate in cardiac fibrosis. The study provides new insight supporting further investigation into the molecular mechanisms, biomarkers and treatment of cardiac fibrosis.

## Data Availability Statement

The data presented in the study are deposited in the PubMed repository, accession number: PRJNA797118.

## Ethics Statement

The studies involving human participants were reviewed and approved by all work with human samples conform to the Declaration of Helsinki, and procedures were approved by the Ethics Committee of Bengbu Medical College and Tianjin First Center Hospital. Informed consent to participate was given by the patient before surgery. The patients/participants provided their written informed consent to participate in this study.

## Author Contributions

YJ and CS: study design, manuscript writing, draft, review, and editing. XZ, TW, XQ, and IA: performed the experiments. NZ, YC, and RW: statistics and draft writing. All authors read and approved the final manuscript.

## Funding

This work was supported by grants from the National Natural Science Foundation of China (81800214), Science foundation for outstanding youth of the first affiliated hospital of Bengbu Medical College (2021byyfyjq02), Excellent Young Talents Fund Program of Higher Education Institutions of Anhui Province (gxyqZD2016167), Key Research and Development Projects of Anhui Province (1804h08020280), and Graduate Scientific Research Innovation Program of Bengbu Medical College (Byycx21077).

## Conflict of Interest

The authors declare that the research was conducted in the absence of any commercial or financial relationships that could be construed as a potential conflict of interest.

## Publisher's Note

All claims expressed in this article are solely those of the authors and do not necessarily represent those of their affiliated organizations, or those of the publisher, the editors and the reviewers. Any product that may be evaluated in this article, or claim that may be made by its manufacturer, is not guaranteed or endorsed by the publisher.

## Supplementary Material

Supplementary Figure 1 **(A)** IF staining detected Vimentin in CFs. Alexa Fluor 594 was used to detect Vimentin, and DAPI was used to stain nuclei. **(B)** CCK8 revealed that the cell proliferation ability was significantly higher under the concentration of 1 × 10^−7^ mol/L at 24 and 48 hours, respectively. Each data point was obtained from three replicate experiments.

Supplementary Figure 2 The enrichment analysis of DEGs in ChIP-seq by DAVID and Metascape. The top 5 GO terms were cell morphogenesis involved in neuron differentiation, regulation of neuron projection development, and axon development. The top 5 KEGG pathways were the phospholipase D signaling pathway, cholinergic synapse, and calcium signaling pathway.

Supplementary Figure 3 **(A)** The hub gene network constructed via cytoHubba_Degree for Ang II vs. CFs. **(B)** The hub gene network constructed via cytoHubba_MCC for Ang II vs. CFs. **(C)** The hub gene network constructed via cytoHubba_MNC for Ang II vs. CFs. **(D)** The hub gene network constructed via cytoHubba_EPC for Ang II vs. CFs.

Supplementary Figure 4 **(A)** The hub gene network constructed via cytoHubba_Degree for CPI vs. CFs. **(B)** The hub gene network constructed via cytoHubba_MCC in for CPI vs. CFs. **(C)** The hub gene network constructed via cytoHubba_MNC for CPI vs. CFs. **(D)** The hub gene network constructed via cytoHubba_EPC for CPI vs. CFs.

Supplementary Table 1 Primer sequences used for ChIP-qPCR.
