# Supplementary figures and images for: Transcriptomic and ChIP-seq Integrative Analysis Identifies KDM5A-Target Genes in Cardiac Fibroblasts
Source: Front Cardiovasc Med. 2022 Jul 1;9:929030. doi: 10.3389/fcvm.2022.929030 (PMC9283924; doi:10.3389/fcvm.2022.929030)

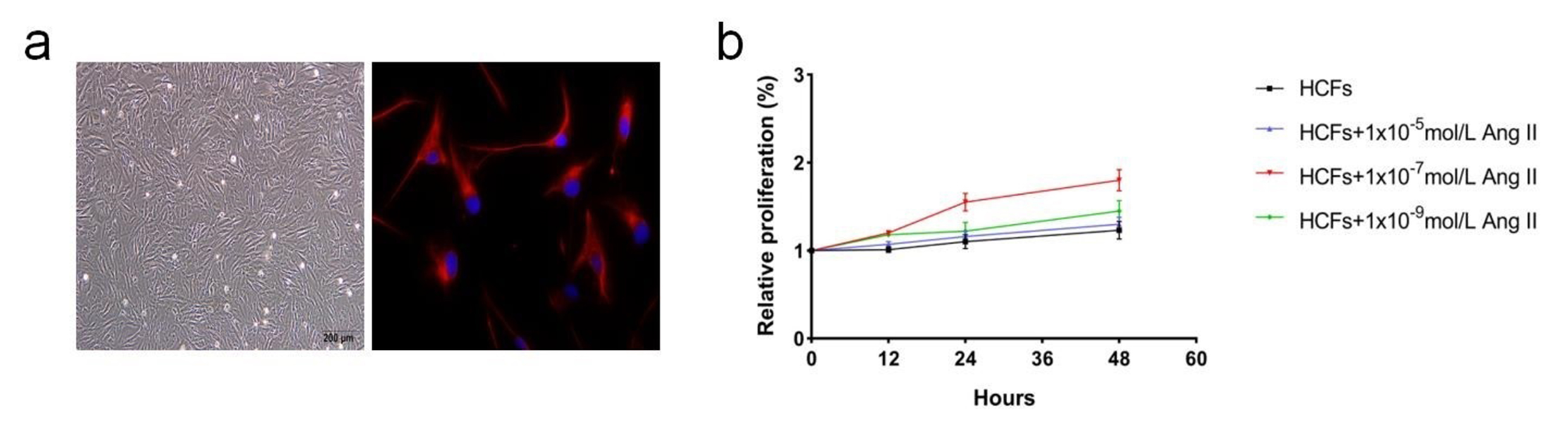

Supplement: Supplementary Figure 1 — (A) IF staining detected Vimentin in CFs. Alexa Fluor 594 was used to detect Vimentin, and DAPI was used to stain nuclei. (B) CCK8 revealed that the cell proliferation ability was significantly higher under the concentration of 1 × 10^−7^ mol/L at 24 and 48 hours, respectively. Each data point was obtained from three replicate experiments. [file Image_1.JPEG]

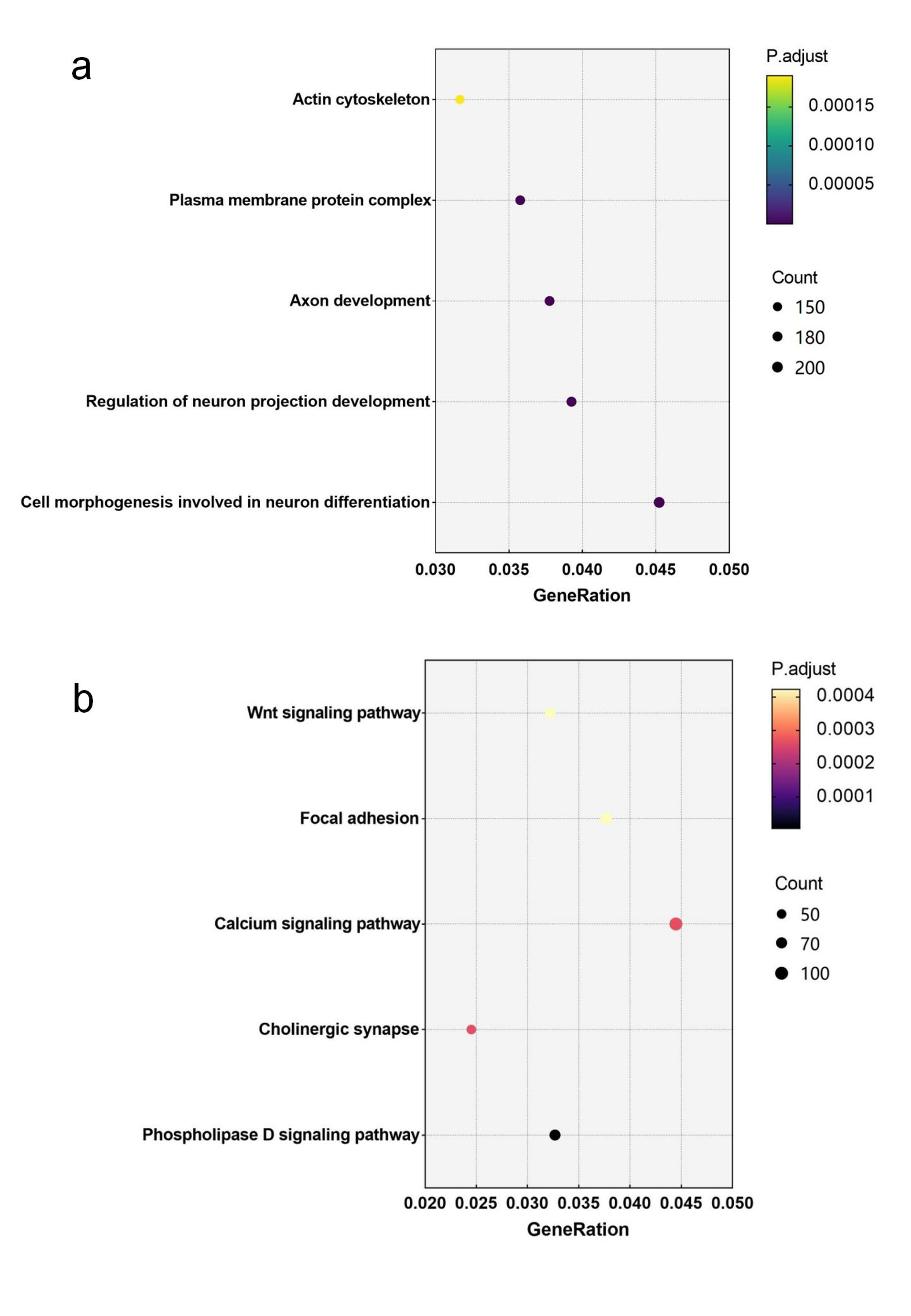

Supplement: Supplementary Figure 2 — The enrichment analysis of DEGs in ChIP-seq by DAVID and Metascape. The top 5 GO terms were cell morphogenesis involved in neuron differentiation, regulation of neuron projection development, and axon development. The top 5 KEGG pathways were the phospholipase D signaling pathway, cholinergic synapse, and calcium signaling pathway. [file Image_2.JPEG]

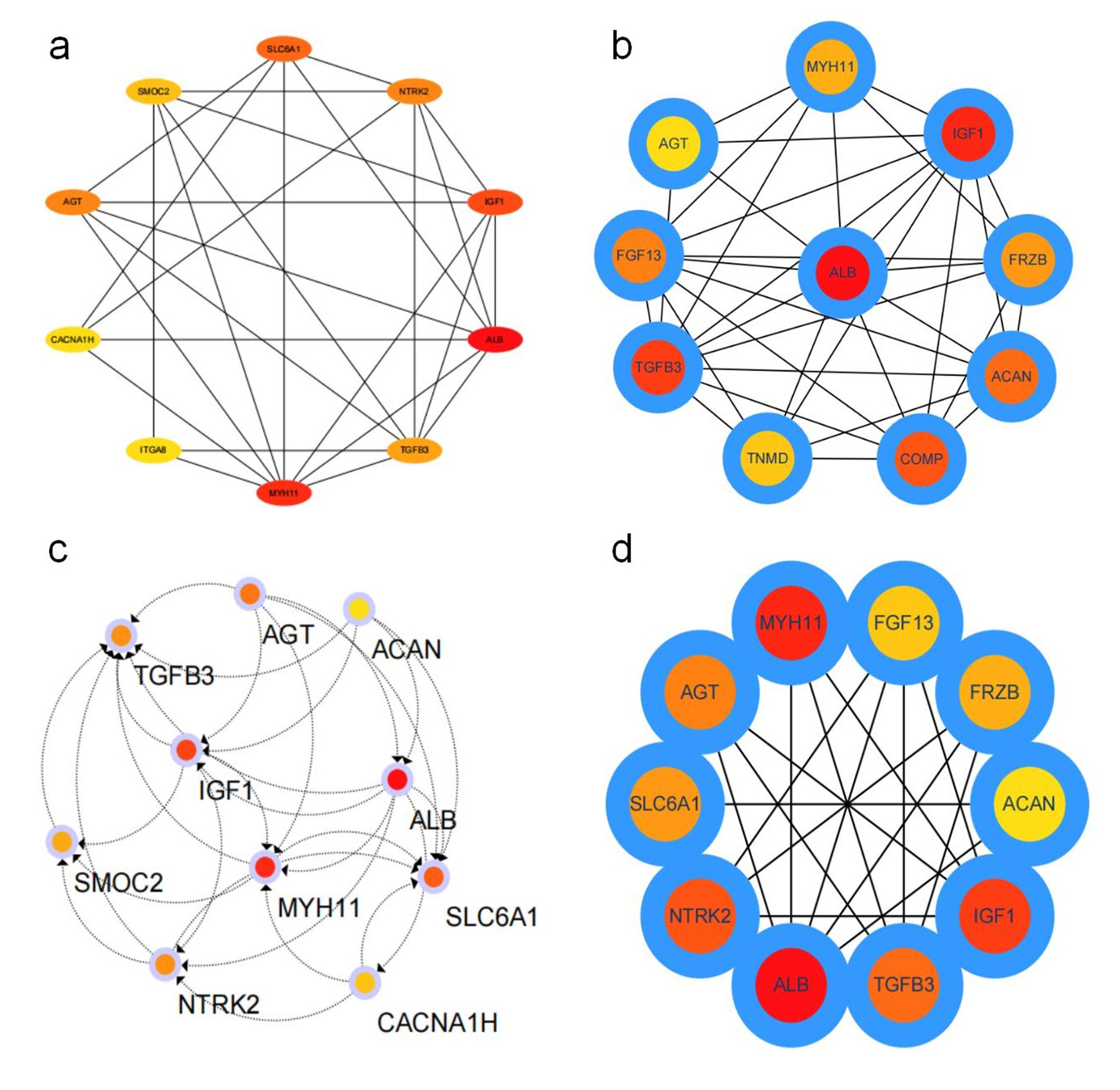

Supplement: Supplementary Figure 3 — (A) The hub gene network constructed via cytoHubba_Degree for Ang II vs. CFs. (B) The hub gene network constructed via cytoHubba_MCC for Ang II vs. CFs. (C) The hub gene network constructed via cytoHubba_MNC for Ang II vs. CFs. (D) The hub gene network constructed via cytoHubba_EPC for Ang II vs. CFs. [file Image_3.JPEG]

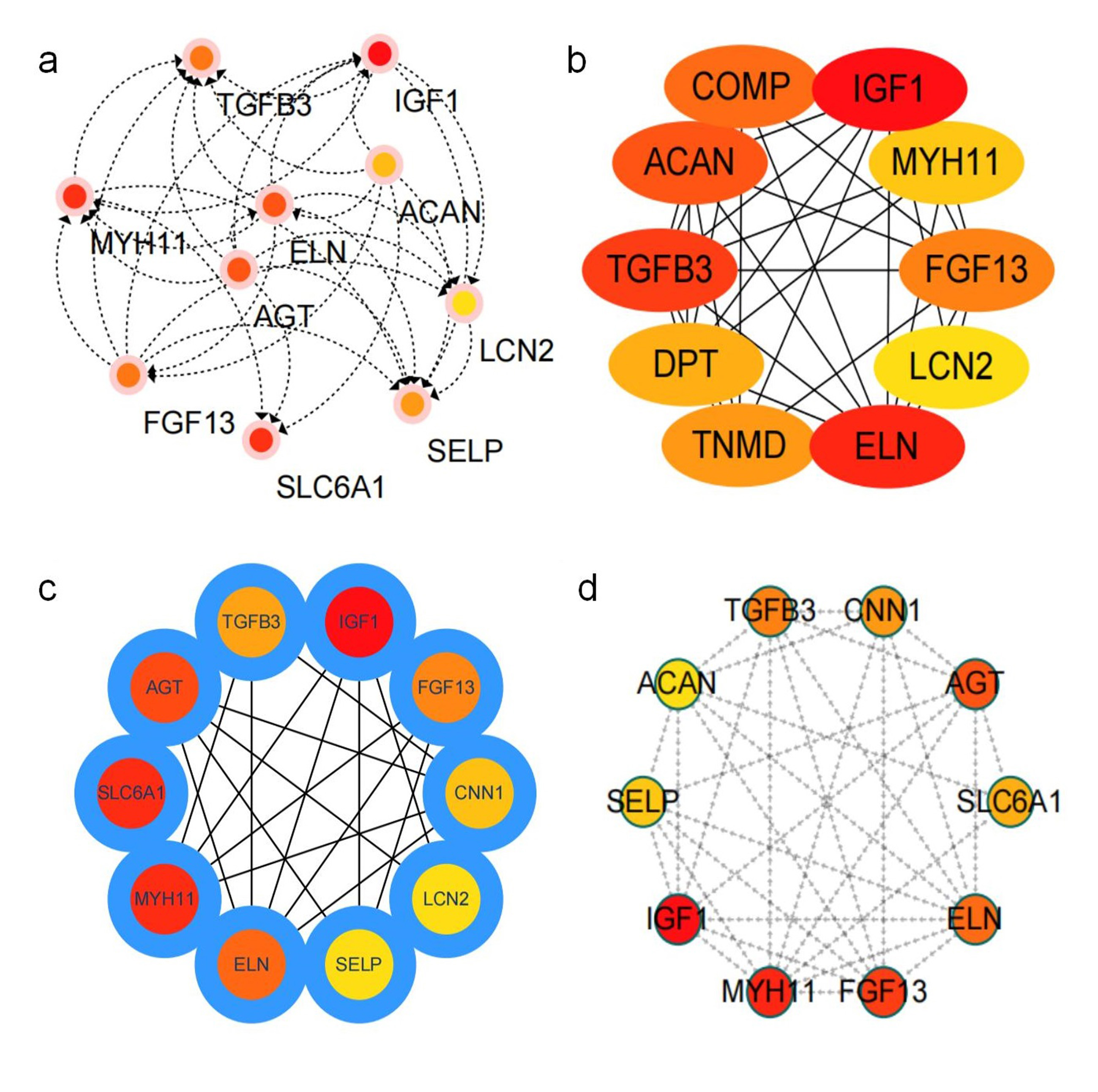

Supplement: Supplementary Figure 4 — (A) The hub gene network constructed via cytoHubba_Degree for CPI vs. CFs. (B) The hub gene network constructed via cytoHubba_MCC in for CPI vs. CFs. (C) The hub gene network constructed via cytoHubba_MNC for CPI vs. CFs. (D) The hub gene network constructed via cytoHubba_EPC for CPI vs. CFs. [file Image_4.JPEG]
