# Supplementary material for: Transcriptomic and ChIP-seq Integrative Analysis Identifies KDM5A-Target Genes in Cardiac Fibroblasts
Source: Front Cardiovasc Med. 2022 Jul 1;9:929030. doi: 10.3389/fcvm.2022.929030 (PMC9283924; doi:10.3389/fcvm.2022.929030)
Supplement: Supplementary Table 1 — Primer sequences used for ChIP-qPCR. [file Table_1.Doc]

Supplementary table 1. Primer sequences used for ChIP-qPCR

|  | Primer sequence | |
| --- | --- | --- |
|  | Forward primer | Reverse primer |
| IGF1 | TATGGGGGATGGGAGAGCAA | CCCGAGTGCTGTCTTCCAAT |
| MYH11 | TTAGGCGAGGAATCCTTGGG | TCCAAGGAGAAAGTGGGTGTG |
| TGFB3 | CCCTTCAGTTAGTGGCCTGG | CCTCTAGGCCTTTTCCCACG |
| ALB | TCTGAGCAAAAGGGCAGAGG | CCTTGACCTTCACCAGGCAT |
| AGT | GATGCTCCCGTTTCTGGGAA | GTGCAGGGTCGAGTTACACA |
| FGF13 | TCGACTCCCGGACTGAGATT | CTGGTCGCCACCTACACTTT |
| ELN | AGCGAAAGAACAGTCGCAGA | AATTACGAAAGGCCCGGCTC |
